# Septic Arthritis Caused by Streptococcus suis Serotype 5 in Pig Farmer

**DOI:** 10.3201/eid2003.130535

**Published:** 2014-03

**Authors:** Christian Gustavsson, Magnus Ramussen

**Affiliations:** Central Hospital, Kristianstad, Sweden (C. Gustavsson); and Lund University, Lund, Sweden (M. Ramussen)

**Keywords:** Streptococcus suis, septic arthritis, zoonoses, bacteria, serotype, pigs, farmer, Sweden, streptococci

**To the Editor:** *Streptococcus suis* primarily infects pigs, but >700 human infections have been reported (*1*). Cases in human occur mainly in persons who have contact with pigs; these infections are most frequently reported in Southeast Asia (*1*). In humans, *S. suis* most often causes meningitis, but endocarditis, pneumonia, toxic shock–like syndrome, and septic arthritis have also been reported (*1*–*3*). 

*S. suis* is classified into serotypes on the basis of the polysaccharide capsule. Among pigs, many serotypes cause severe infections, but nearly all human cases have been attributed to serotype 2 (*1*,*3*). Other serotypes have been isolated from humans only in a few cases: meningitis caused by serotype 4 (*2*); fatal bacteremia caused by serotype 16 (*4*); sepsis caused by serotype 24 (*5*); bacteremia, meningitis, and endocarditis caused by serotype 14 (*6*–*8*); and spontaneous bacterial peritonitis caused by serotype 5 (*5*). Here, we report a case of septic arthritis caused by *S. suis* serotype 5.

The patient was a 65-year-old pig farmer who had cut his hand at work; he had not noted cases of severe illness among his pigs. He had a history of benign hyperplasia of the prostate gland, and 1 year before the current illness, he received a diagnosis of right-sided coxarthrosis, for which radiographic imaging showed grade II changes, loss of cartilage, and subchondral sclerosis. One week after the patient cut his hand, his right hip became increasingly painful, and he sought treatment at a hospital. On examination, the trochanter major region was tender (not noted at previous examinations), and passive movement of the hip was painful. Blood test results showed a slight elevation of C-reactive protein (CRP), to 31 mg/L (reference <5 mg/L). The symptoms were interpreted as trochanteritis, and treatment with nonsteroidal anti-inflammatory medication was instituted. The next day, the patient returned to the hospital with worsened pain and was admitted. He had a temperature of 37.7°C and a heart rate of 80 beats/min; blood test results showed a leukocyte count of 11.2 × 10^9^ cells/L and CRP of 127 mg/L. Radiologic images of the hip were unremarkable, but ultrasonography-guided joint puncture showed pus and blood in the synovial fluid. Cultures were secured, and gram-positive cocci in short chains were noted in all blood culture bottles and in the synovial fluid culture. Treatment with intravenous cefotaxim was started. 

Microbiological diagnosis of *S. suis* infection was made on the basis of colony morphology, a weak reaction with Lancefields anti-D antiserum, and a score of 2.31 according to matrix-assisted laser desorption/ionization–time of flight mass spectrometry (Biotyper version 3.0 software; Bruker Daltonics, Bremen, Germany). On the fourth day after admission, treatment was changed to benzylpenicillin (3 g 3×/d). The pain from the hip gradually declined, and CRP peaked at 337 mg/L on the fifth day after admission. On the seventh day after admission, treatment was changed to oral penicillin (2 g 3×/d) and was continued for 6 weeks. 

At follow-up 6 months after the initial illness onset, the impairment in the patient’s hip movement had worsened. Radiologic imaging showed necrosis of the femoral head, and the patient underwent total hip replacement surgery. During surgery, no signs of synovitis were noted, and 5 intraoperative cultures were negative. The procedure was completed without complications, and the patient’s symptoms resolved.

The *S. suis* isolate from the patient was determined to be serotype 5 by Statens Serum Institut (SSI; Hillerød, Denmark) by agglutination with latex beads and type-specific serum and by microscopic determination of capsule swelling with type-specific serum (SSI Diagnostika, Hillerød, Denmark), according to the manufacturer’s instructions. These methods gave concordant results. Etests (bioMérieux, Solna, Sweden) demonstrated that the isolate was sensitive to all antimicrobial drugs tested; MIC was 0.125 mg/L for cefotaxim and 0.016 mg/L for benzylpenicillin. The isolate was tested for known virulence-associated genes *sly*, *mrp*, and *epf* with PCR, as described (*9*). PCR fragments of predicted sizes were obtained with primer hybridizing to *sly* and *mrp* but not with primers hybridizing to *epf.* A serotype 2 isolate (kindly provided by Susanne Sauer at SSI) was used as a positive control for the *epf* primers.

*S. suis* is an emerging human pathogen, but reports of human infections caused by serotypes other than serotype 2 remain scarce. This case demonstrates that *S. suis* of serotype 5, which is a serotype routinely isolated from deceased pigs (*10*), can cause invasive infections in humans. The course of the described infection was relatively favorable, and the patient did not show signs of a systemic inflammatory response syndrome or of meningitis. Preexisting osteoarthritis of the right hip might have had diminished local defenses, thereby enabling colonization of the hip area by bacteria that had entered the bloodstream through the wound on the patient’s hand. The isolate we recovered possessed *sly* and *mrp* genes, which encode the virulence-associated suilysin and muraminidase-released proteins, but clearly other factors are also of importance for determining the virulence of individual *S. suis* isolates.
