# Correction: Vol. 20, No. 3

**DOI:** 10.3201/eid2101.C12101

**Published:** 2015-01

**Keywords:** errata, erratum, correction

The name of author Magnus Rasmussen was misspelled in the article Septic Arthritis Caused by *Streptococcus suis* Serotype 5 in Pig Farmer (C. Gustavsson, M. Ramussen). The article has been corrected online (http://wwwnc.cdc.gov/eid/article/20/3/13-0535_article).

